# Enhanced Dissolution Rate of Tadalafil Nanoparticles Prepared by Sonoprecipitation Technique: Optimization and Physicochemical Investigation

**Published:** 2017

**Authors:** Rayehe Teymouri Rad, Seyed Alireza Mortazavi, Alireza Vatanara, Simin Dadashzadeh

**Affiliations:** a *Pharmaceutics Department, School of Pharmacy, Shahid Beheshti University of Medical Sciences, Tehran, Iran.*; b *Pharmaceutics Department, School of Pharmacy, Tehran University of Medical Sciences, Tehran, Iran.*; c *Studentsʼ Research Committee, Shahid Beheshti University of Medical Sciences, Tehran, Iran.*

**Keywords:** Tadalafil, Poorly water-soluble drug, Antisolvent precipitation-ultrasonication method, Nanocrystals, Dissolution enhancement

## Abstract

Nanocrystals of tadalafil, a poorly water-soluble drug, were successfully prepared by sonoprecipitation technique for improving the solubility and dissolution rate. Tween 80 was selected as an efficient surfactant to inhibit aggregation in stabilization of drug nanocrystals. Response surface methodology based on central composite design (CCD) was utilized to evaluate the formulation factors affecting the size of nanosuspensions. Under optimum conditions, relatively spherical nanocrystals with a mean particle size of 358.47 ± 11.95nm were obtained. FTIR analysis indicated that the precipitated nanoparticles had the same chemical structure as the raw tadalafil. By DSC analysis, no substantial crystalline change was found in the nanocrystals compared with the unprocessed drug. In addition, the dissolution rate of the processed tadalafil nanocrystals in 120 min was significantly increased (3.61-fold) as compared to that of the raw material. Therefore, it was concluded that the sonoprecipitation technique could be a simple and useful technique to prepare poorly water-soluble drug particles with reduction in particle size, a narrow particle size distribution and enhanced dissolution properties.

## Introduction

**Figure 1.**
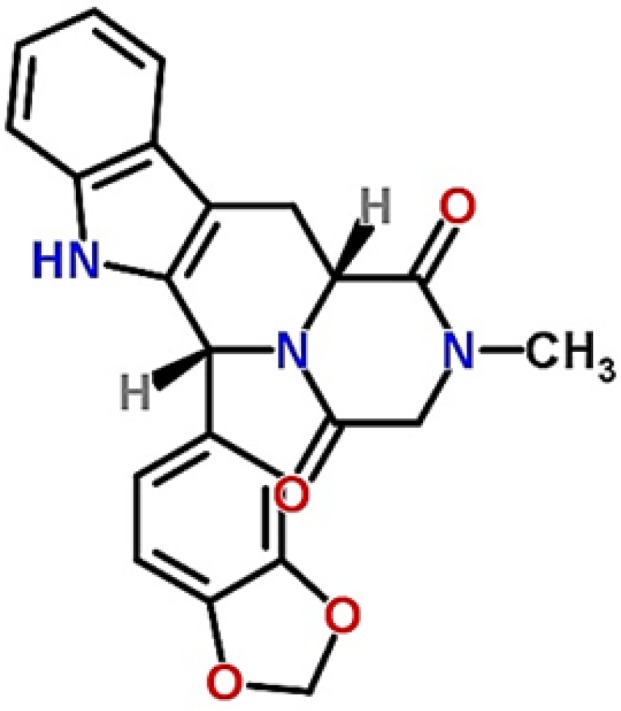
Chemical structure of tadalafil (25).

Tadalafil is a potent and selective cyclic guanosine monophosphate specific type (V) phosphodiesterase inhibitor used for the treatment of erectile dysfunction, benign prostatic hyperplasia and pulmonary arterial hypertension (1-3). The chemical structure of tadalafil is shown in Figure 1. Longer duration of action (approximately 36 h) and minimized potential for vision abnormalities are the most important advantageous of tadalafil in comparison with other drugs in this pharmacological category (4). Since it belongs to class II of classification system, poor aqueous solubility and wettability is an important issue and in spite of good permeability, its bioavailability is limited by solubility and dissolution rate, leads to variability in blood concentrations and irreproducibility in clinical responses (5). Different groups have utilized various formulation strategies for improving the solubility and dissolution rate of tadalafil that the most reported ones include preparation of solid dispersion by using water soluble polymers and copolymers (such as poly(vinyl pyrrolidone) k 30, poly(ethylene glycol) 6000, poloxamer 188 and 407 (6), formation of inclusion complex with ß-cyclodextrins and microporous silica (7, 8), the use of amorphization methods (such as vitrification, cryogenic grinding, ball milling, spray drying and freeze drying) (9). However, to the best of our knowledge a few studies have been conducted in the field of nanoparticles preparation by antisolvent precipitation-ultrasonication method.

Drug particle size reduction has emerged as an effective and versatile option for surmounting solubility issue (10, 11). According to Noyes-Whitney equation, dissolution rate increases when the surface area increases by reducing the size to nanometer (12). 

Nanosuspension or nanocrystal suspension is a pure particulate system that is composed of submicron (average particle size in the range of 200-600 nm) colloidal dispersion with a surfactant as the stabilizer (13). As the first time, in 2000, nanosuspensions have been commercialized in the pharmaceutical market. Increased saturation solubility, increased adhesiveness to surfaces/cell membranes and increased dissolution rate are special properties of nanosuspensions 

(14). Techniques used to produce drug nanosuspensions can be classified into two major groups: top-down and bottom-up technologies (15). One of the most bottom-up promising techniques is nanoprecipitation that is cost-effective, rapid to perform and suited for scaling up (16, 17). 

In this method, nanoparticles could be formed in different ways such as pH controlled precipitation, antisolvent precipitation with or without surfactant and sonoprecipitation (18). Sonoprecipitation, a combination of antisolvent precipitation and ultrasoication, is an effective method of controlling the nucleation and crystallization process (19). 

Ultrasound amplifies the mass transfer when it propagates through a liquid medium, and initiates an important phenomenon known as cavitation. Furthermore, it increases micro-mixing, reduces particle growth and agglomeration, so it is possible to obtain particles with uniform size distribution (20). In a study, Xia *et al.* (21) succeed to prepare nitrendipine nanosuspensions with diameter of about 209 ± 9 nm with an enhanced dissolution rate and bioavailability. Also, the same results were found in the preparation of carvedilol (22), itraconazole (23), clarithromycin, cefixime, glipizide nanosuspensions by other groups. In this technique, in addition of the type of antisolvent and stabilizers, process parameters such as the precipitation temperature, the power input and the time length of ultrasonication play an important role (21). 

Formulation parameters such as drug concentration, stabilizer concentration and antisolvent to solvent ratio also need to be optimized because of their important influence on supersaturation degree and nucleation rate (24).

The aim of this study was to prepare tadalafil nanosuspension by sonoprecipitation technique and optimize process parameters in order to enhance solubility and dissolution rate of the drug.

*Materials*

The raw tadalafil was purchased from Dr. Reddy’s Pharmaceutical Co., Ltd., India. Tween80, sodium hydroxide (NaOH), monobasic potassium phosphate (KH2PO4), methanol, ethanol, dimethyl sulfoxide (DMSO), acetone and high-performance liquid chromatography (HPLC) grade acetonitril were obtained from Merck, Germany. Deionized water was prepared with Millipore water purification system, Germany.

*Methods*

*Preparation of tadalafil Nanocrystals by Sonoprecipitation*

**Figure 2:**
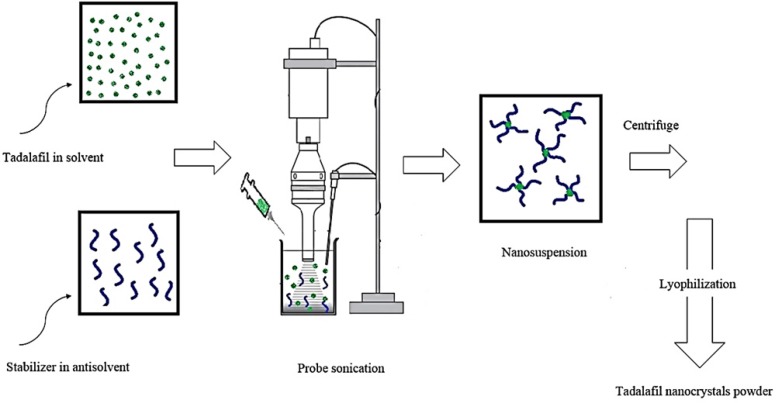
Experimental process to prepare the tadalafil nanoparticles.

Tadalafil nanosuspensions were prepared through sonoprecipitation technique. The experimental process for the preparation of tadalafil nanosuspension is illustrated in Figure 2. In a typical procedure, combination of two organic solvents, acetone: DMSO (88:12 v/v), was selected as water-miscible solvent and deionized water was used as antisolvent. Tadalafil coarse powder was completely dissolved in the organic phase and then was filtered through a syringe filter, PTFE membrane with pore size of 0.45 µm (Simplepure, USA) to remove the possible particulate impurity. A range of surfactants and polymers (such as hydroxypropyl methylcellulose in grades of 6, 15 and 50 cp, poly (ethylene glycol) 400 and 6000, polyoxyl 40 stearate, poloxamer 188 and 407, poly (vinyl pyrrolidone) k30, poly (vinyl alcohol), sodium carboxymethyl cellulose, Tween 80 and sodium lauryl sulfate) were screened as stabilizer and finally Tween 80 was selected in accordance with the size criteria (data not shown). The antisolvent phase was prepared by dispersing a stabilizer in distilled water that was cooled to 5±1 °C in an ice-water bath and treated with an ultrasonic probe (Hielscher UP400S, 400W, 24 kHz, Germany) at power input of 280 W and a cycle of 0.5 per second. In the next step, precipitation initiated by drop-wise adding of drug solution phase within 5 min. As the nanosuspension emerged, size and polydispersity index (PDI) were evaluated. Then, the obtained nanosuspensions were concentrated by centrifugation at 16000rpm for 50 min using an ultracentrifuge (3-30K, Sigma, Germany) and washed three times with deionized water. Furthermore, the content of drug was determined in supernatant by HPLC analysis for calculation of yield. Finally, the obtained nanoparticle residue was frozen at -80°C for 24 h and subsequently lyophilized for 48 h at a temperature of 58 °C under vacuum by using freeze dryer (Alpha 1-2 LDplus, Christ, Germany). The dried powder was then collected and stored in air tight container for further use.

*Experimental design*

Response surface methodology based on central composite design was employed for evaluation of the formulation factors *i.e*., drug concentration (A), stabilizer concentration (B), antisolvent to solvent volume ratio (C) and mean particle size (y) was assessed as the response. The details of design are shown in Table 1. Based on the results of preliminary experiments, the experimental range of each variable was chosen. The experiments were designed using Design-Expert^®^ software (ver. 7.0.0, stat-ease^®^, USA). To reduce systematic errors, the experiments were completely randomized.

By Analysis of collected data for responses, the relationship linking the main factors and their interactions to the responses were determined and presented as a general form in the following Equation (1).

Equ 1 Y= intercepts+∑maineffects+∑interactions

A quadratic model Equation (2) was fitted to the response using the Design-Expert^®^ software.

$y=\beta_{0}+\beta_{1}A+\beta_{2}B+\beta_{3}C+\beta_{11}A^{2}+\beta_{22}B^{2}+\beta_{33}C^{2}+\beta_{12}\mathrm{AB}+\beta_{31}\mathrm{AC}+\beta_{23}\mathrm{BC}\ldots .$

Equ 2

Where y represents the predicted response, A, B and C represents the independent variable and ß represents the coefficient. The three dimension (3D) response surface graphs were plotted using origin 7.0 software according to the equation.

*Physicochemical Characterization of tadalafil nanocrystals*

*Particle size and zeta potential analysis*

The mean particle size (z-average), polydispersity index (PDI) and zeta potential were determined by photon correlation spectroscopy (PCS) using Zeta-sizer (Nano-ZS, Malvern Instruments, UK). The real refractive index and the imaginary refractive index were set at 1.76 and 0.01, respectively. The z-average and PDI values were obtained by averaging of three measurements. Before the measurement, a small aliquot of nanosuspensions was diluted with 5 mL of deionized water to have a suitable scattering intensity and then sonicated to create a homogenous suspension.

*Yield of nanoprecipitation process*

Nanosuspensions were centrifuged (3-30K, Sigma, Germany) at 16000 rpm for 50 min. The concentration of dissolved tadalafil in the supernatant was determined using a reverse phase high performance liquid chromatography (RP-HPLC) (Smart line 1000 pump, Smart line Diode array 2800 UV detector, Knauer, Germany) as was reported by Cheng and *et a.l* (26). EZ CHROM software was used to record and evaluate the data collected during and following chromatographic analysis. The chromatographic separation was accomplished using a Perfectsil target C_18_ column (150 ×4.6 mm, 5µm) (ODS-3, MZ, Germany) protected by a guard column (10×4.6mm) which was packed with the same C_18_ material. Acetonitrile and 20 mm phosphate buffer (pH 7.0) in a ratio of 40:60 was used as the mobile phase. The column was maintained at 25 °C and equilibrated for 60 min with the analytical mobile phase before injection. The injection volume was 20 µL, and the mobile phase was pumped isocratically at a flow rate of 1.0 mL/min. The eluent was analyzed at 283 nm and the retention time of the drug was 9.45 min. The yield of the process was calculated using the Equation 3. 

Equ 3 $$(\%)\mathrm{Yield}=\frac{\left(\mathrm{Total drug}-\mathrm{Dissolved drug}\right)}{\mathrm{Total drug}}\times 100$$

*Particle morphology*

The morphology of tadalafil and the tadalafil nanocrystals was evaluated through field emission scanning electron microscope (S-4160, Hitachi, Japan). Prior to analysis, the samples were diluted with ultra-purified water to obtain a suitable concentration. Then, the samples were spread on a sample holder, dried under vacuum and eventually coated with gold.

*Fourier transformed infrared spectroscopy (FTIR)*

FTIR technique was applied to determine the molecular structures of raw tadalafil and the interaction between stabilizer and drug nanocrystals. FTIR spectra were recorded by FTIR spectrometer (Nicolet Magna-IRTM, USA) within the spectral region of 400 and 4000cm^−1^ at a resolution of 2 cm^−1^. The IR spectra were obtained in a KBr disc.

*X-ray powder diffraction (XRPD)*

The crystal forms of the samples were detected using a powder X-ray diffractometer (IPDS II, STOE, Germany). The current and voltage using Cu Kαl radiation were 30 mA and 40 kV, respectively. The obtained data were typically collected from 1 to 40 with a step size of 0.06 at a rate of 1/s. the output is given as intensity versus 2θ.

*Differential scanning calorimetry (DSC)*

The thermal analysis was performed using differential scanning calorimeter (DSC-60, Shimadzu, Japan). Approximately, 3 mg of each samples was placed in an aluminum pan. The heating and cooling scans were performed from -10 °C to 320 °C at the heating and cooling rates of 10 °C/min in a dry N_2_ atmosphere. An empty aluminum pan was used as a reference. The melting temperature and enthalpy were calculated from the DSC thermograms.

*Dissolution rate study*

The dissolution rate experiments of raw tadalafil and tadalafil nanocrystals were carried out according to the USP 32 apparatus II (paddle) method. Phosphate buffer (pH 7.4) was used as the dissolution medium. The stirring speed and the bath temperature were 100 rpm and 

37.0 ± 0.5 °C, respectively. Unprocessed tadalafil (2 mg) and tadalafil nanocrystals (containing 2 mg tadalafil) were added to 900 mL of dissolution medium. Then, aliquots equivalent to 5 mL were withdrawn after 10, 20, 30, 45, 60, 90 and 120 min and immediately replaced with the same volume of phosphate buffer. All of samples were passed through a 0.45 µm syringe filter and injected into the HPLC system for analysis of drug concentration. Furthermore, the obtained dissolution profile data of the raw tadalafil 

and tadalafil nanocrystals were evaluated and compared using the dissolution efficiency (DE%) in 30 and 90 min. The measurements were repeated three times. This concept was proposed by Khan and Rhodes (27) and is defined as follows:

Dissolution Efficiency DE%=∫0ty.dty100t×100

Equ. 4

Where y is the percent of drug dissolved at any time t, y_100 _denotes 100% dissolution, and the integral represents the area under dissolution curve between time zero and t.

*Saturation solubility*

The saturation solubility of raw tadalafil and tadalafil nanocrystals were evaluated in two mediums: deionized water and phosphate buffer (pH=7.4) at 25 °C. Excess amounts of lyophilized powder was dispersed in 10 mL of medium and placed on a stirrer for 48 h to ensure that the solubility equilibrium had been reached. The samples were centrifuged and the resulting supernatant was filtered and then analyzed by HPLC. The measurements were repeated three times.

*Statistical analysis*

The reported data represented the mean value ± standard deviation (SD). Significance of difference was evaluated using Student t-test and one-way ANOVA at the probability level of 0.05 using SPSS 19 for Windows (SPSS, Chicago, IL, USA) and Design-Expert® software (ver. 7.0.0, stat-ease^®^, USA).

## Results and Discussion

Tadalafil nanocrystals were prepared and optimized using sonoprecipitation technique. In this method, introduction of the drug solution to the antisolvent generates high supersaturation and results in fast nucleation rate to produces a large number of nuclei, which reduces the solute mass for subsequent growth. Growth of nucleating crystals can be restricted in the presence stabilizer (surfactant or polymer) through steric or electrostatic mechanisms (17). In the case of Tween 80 with CMC of about 1.4 $\times 10^{-2}$moL/l (0.6%), hydrophobic moieties that adsorb on the hydrophobic drug particles, prevents crystals from aggregation due to the steric repulsion (28, 29). On the other hand, during the negative-pressure period of the ultrasound irradiation, cavitational bubbles are formed that cause powerful shock wave because of very rapid collapse. In fact, sonoprecipitation could act in two ways as induction of primary nucleation in particle free solutions and shortening the induction time between supersaturation and the onset of nucleation and crystallization (30).

*Formulation optimization and statistical analysis*

A central composite design (CCD) is a factorial or fractional factorial design with center points, augmented with a group of axial points that let you estimate curvature. The effect of a wide range of dependent variables is predictable by CCD. Drug concentration, stabilizer concentration and antisolvent/solvent volume ratio were independent variables in the preparation of tadalafil nanosuspension; while, particle size was studied as the dependent variable in a total of 20 experiments (Table 2). The individual and interactive effects of various variables were studied by carrying out the process at different levels of all factors.

The particle size of nanoparticles was in the range of 254.3-1138.0 nm and the quadratic model was the best fitted on the data. The obtained model was validated using an ANOVA. As presented in Table 3, the most effective parameters on the size of nanoparticles was stabilizer concentration (B) with the F value of 16.72 (*P *<0.05) and drug concentration (A) with the F value of 5.47 (*P *<0.05).

According to the results, a two second-order polynomial representing particle size was generated, which is shown below in terms of coded factors (Equation 5).

Equ 5 $$\mathrm{Particle}\mathrm{size}\left(\mathrm{nm}\right)=-12.66-0.74A+1.29B+0.17C+1.42A^{2}-0.79B^{2}+0.095C^{2}+0.44\mathrm{AB}+0.36\mathrm{AC}+0.038\mathrm{BC}$$

The R^2^ value was 0.9096. Thus, the best formulation can be predicted for tadalafil nanosuspensions by the fitted equation. The predicted R-squared (0.5471) was in reasonable agreement with the adjusted R-squared (0.7062). Adequate Precision measures the signal to noise ratio. Since a ratio greater than 4 is desirable, our ratio of 11.67 indicates an adequate signal. This model can therefore be used to conduct the design.

**Figure 3:**
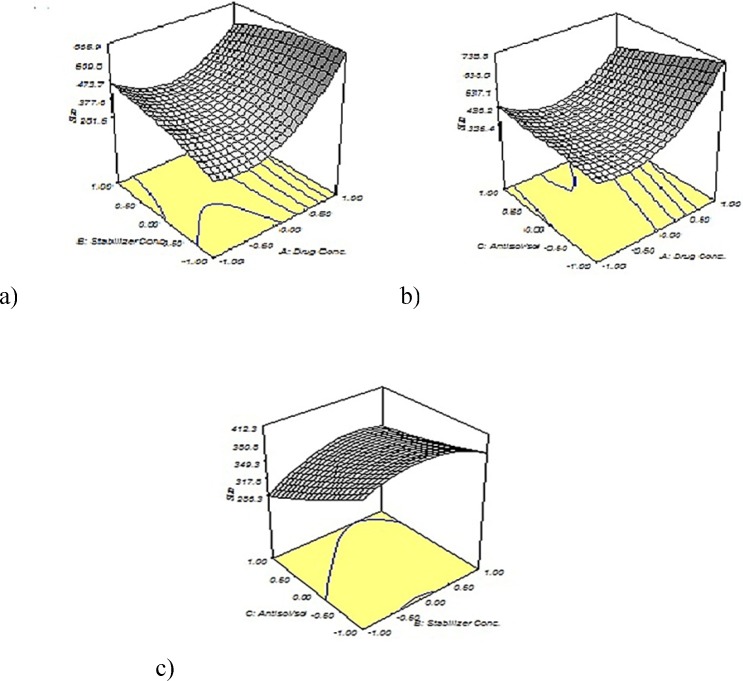
Response surface for particle size. (a) The antisolvent/solvent volume ratio was fixed at 22.5. (b) The stabilizer concentration was fixed at 0.2 % w/v. (c) The drug concentration was fixed at 20 mg/ml

As shown in Figures 3a and 3b, the particle size increased significantly with increasing the concentration of tadalafil. This phenomenon could be interpreted by considering this fact that under the appropriate concentration, higher supersaturation produces smaller particles. At first by increasing the drug concentration; higher supersaturation level creates and a large number of nuclei forms that result in small particle size. Although, at higher level of drug concentration condensation and/or coagulation promotes that accelerates crystal growth so large particle size is expected. (21, 31, 16). In Figure3c the particle size illustrates an initial increase and then a slight decrease with increasing Tween 80 concentration. Also, an ascending trend in particle size with increasing stabilizer concentration can be observed in Figure 3a In point of fact, when the Tween 80 concentration was optimum, it could coat the surface of the drugs effectively and inhibit particles growth by steric stabilization. However, by increasing the Tween 80 level, drug solubility in the stabilizer solution increase and particle size from Ostwald ripening increase, too (22, 32). Figures 3 b and 3 c show two various effects of increasing the antisolvent/solvent volume ratio on the average tadalafil particle size that it arises from interaction between different variables. Generally, the higher degree of supersaturation causes the smaller particle size of crystals. Under the same concentration of tadalafil, the solubility of tadalafil in the mixed solution decreases with the increase in the proportion of antisolvent. This caused the increase of supersaturation degree, which resulted in decreased particle size during re-crystallization. Although, the particle agglomeration would occur due to the high nucleus concentration of drug, when volume ratio exceeded (33, 34).

Desirability function was used for optimization to obtain the levels of formulation parameters while the size of particles is minimized. The optimum formulationconditions were as drug concentration of 10 mg/mL, stabilizer concentration of 0.236 % w/v and antisolvent/solvent volume ratio of 15:1. The nanosuspensions prepared in the optimized formulation yielded a mean particle size of 358.47 ± 11.95 nm, which was in good agreement (± 10%) with the value predicted by the quadratic model (336.8 nm). The results confirmed that the model was effective for predicting the impact of formulation composition on the particle size reduction of tadalafil nanosuspensions.

**Figure 4.**
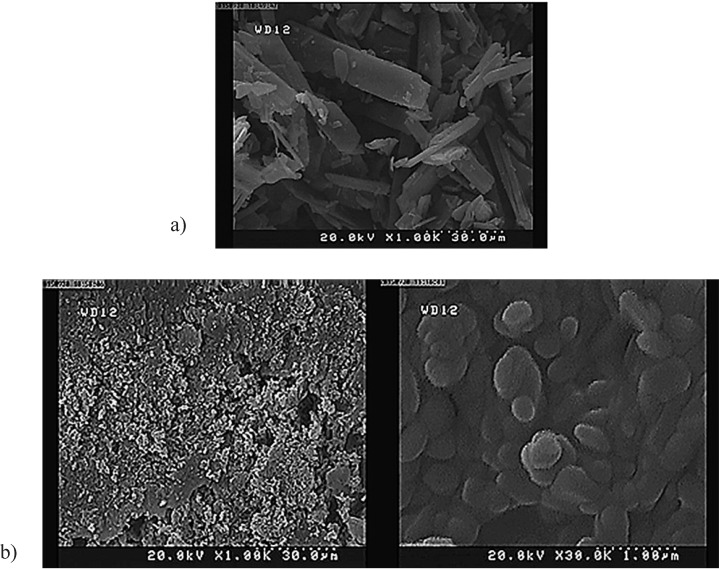
FE- SEM image of (a) raw tadalafil and (b) tadalafil nanocrystals under the optimum condition

**Figure 5:**
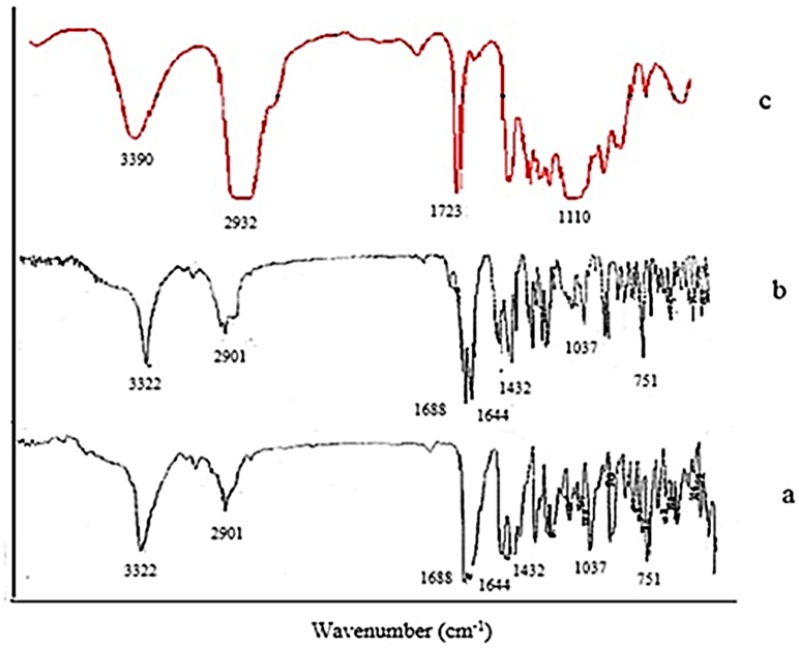
FTIR spectra of (a) raw tadalafil, (b) tadalafil nanocrystals and (c) Tween 80

**Figure 6:**
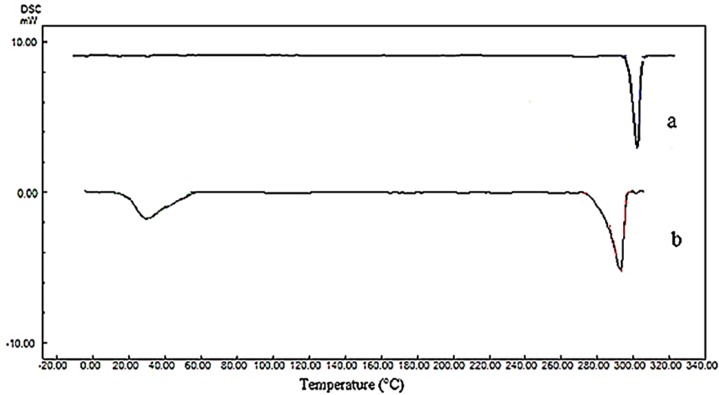
DSC thermograms of (a) raw tadalafil and (b) tadalafil nanocrystals

**Figure.7:**
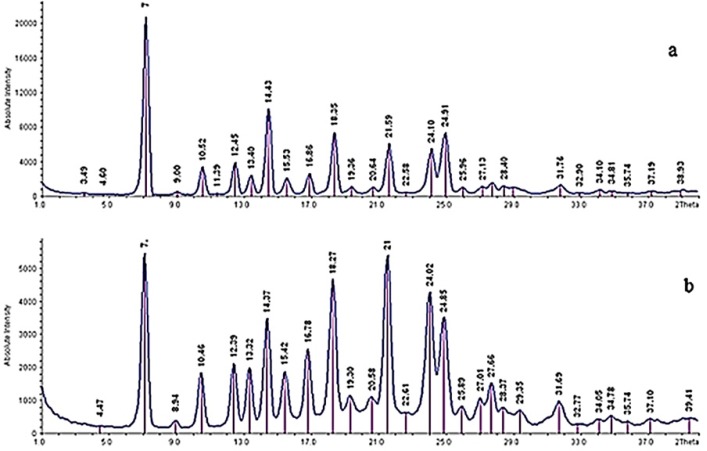
XRPD patterns of (a) raw tadalafil and (b) tadalafil nanocrystals

**Figure.8:**
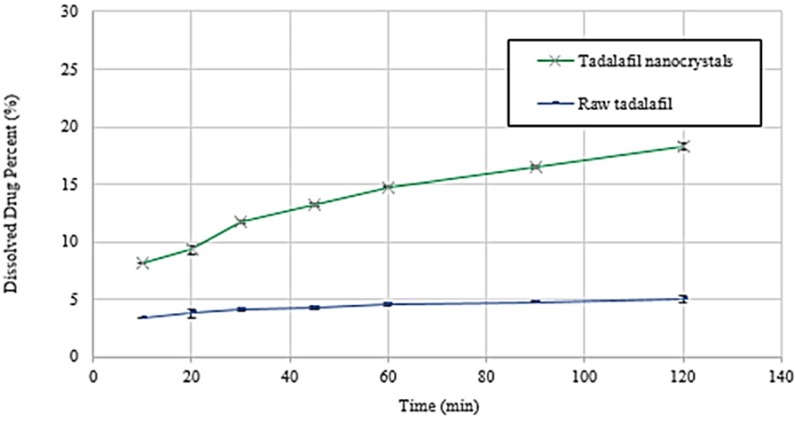
Dissolution profiles of raw tadalafil and tadalafil nanocrystals in phosphate buffer (pH=7.4) medium at 37.0±0.5 °C. Each value represents the mean ± SD (n=3

**Figure 9:**
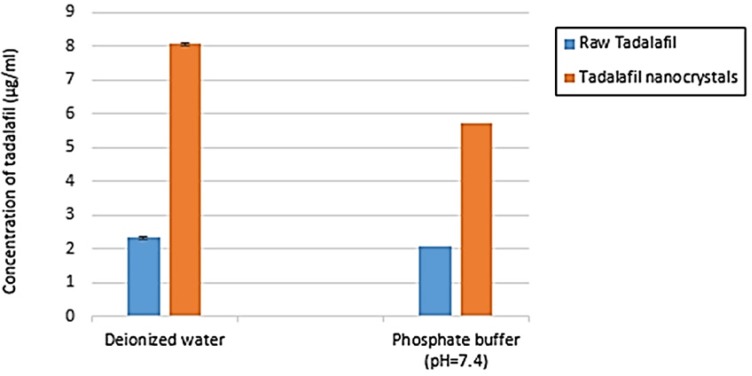
The result of saturation solubility of raw tadalafil and tadalafil nanocrystals in two medium: deionized water and phosphate buffer (pH=7.4) at 25 °C. Each value represents the mean ± SD (n=3

**Table 1:** Numeric variables and their levels investigated in the CCD for preparation of tadalafil nanosuspensions

Variables	Code	Range and levels				
		-1.68	-1	0	+1	+1.68
Drug concentration (mg/mL)	A	3.2	10	20	30	36.8
Stabilizer concentration (%)	B	0.032	0.1	0.2	0.3	0.368
Antisolvent/solvent volume ratio	C	9.9:1	15:1	22.5:1	30:1	35.1:1

**Table 2:** Suggested formulation by CCD for preparation of tadalafil nanaosuspention

Run	Levels of factors			
	A	B	C	Size (nm)
1	+1	+1	+1	372.7
2	-1	+1	+1	516.4
3	0	0	+1.68	400.4
4	0	0	0	362.7
5	-1	+1	-1	377.7
6	0	0	-1.68	536.8
7	-1	-1	+1	254.3
8	+1	-1	-1	658.4
9	0	0	0	375.4
10	+1	-1	+1	480.7
11	+1.68	0	0	636.7
12	0	0	0	322.8
13	0	-1.68	0	394.7
14	-1	-1	-1	274.0
15	+1	+1	-1	585.2
16	0	+1.68	0	396.6
17	0	0	0	406.6
18	-1.68	0	0	1138.0
19	0	0	0	345.2
20	0	0	0	387.0

**Table 3:** The contribution and significance of different formulation parameters on particle size

Source	Sum of squares	DF	Mean square	F value	Prob>F	
Model	74.83	9	8.31	6.07	0.0047	Sig
A	7.49	1	7.49	5.47	0.0414	
B	22.88	1	22.88	16.72	0.0022	
C	0.40	1	0.4	0.30	0.5987	
A^2^	28.86	1	28.86	21.09	0.0010	
B^2^	8.98	1	8.98	6.56	0.0283	
C^2^	0.13	1	0.13	0.094	0.7650	
AB	1.53	1	1.53	1.12	0.3151	
AC	1.05	1	1.05	0.77	0.4014	
BC	0.011	1	0.011	8.219 E-003	0.9296	
Residual	13.69	10	1.37			
Lack of fit	3.31	5	0.66	0.32	0.8820	Not sig
Pure error	10.37	5	2.07			
Cor total	88.52	19				

**Table 4:** DSC data of raw tadalafil and processed tadalafil nanocrystals

Raw tadalafil	301.3	-102.16
**Tadalafil nanocrystals**	292.96	-98.61

**Table 5:** Dissolution efficiency data of raw tadalafil and processed tadalafil nanocrystals after 30 and 90 min. Each value represents the mean ± SD (n=3

	DE_30_ (%)	DE_90_ (%)
Raw tadalafil	3.06 ± 0.13	3.81±0.10
Tadalafil nanocrystals	7.99 ± 0.11	11.4±0.09

*Physicochemical characterization of optimized nanocrystals*

*Particle size, zeta potential and yield*

The mean particle size, PDI, zeta potential and yield of three batches of optimized nanosuspensions were 358.47 ± 11.95 nm, 0.237 ± 0.033, -14.3 ± 1.1 mV and 95.62 ± 1.17 %, respectively. 

*Particle morphology*

FE-SEM images of the raw tadalafil and processed tadalafil nanocrystals are shown in Figure 4. The raw tadalafil exhibited acicular crystals and wide particle size distribution (Figure 4a); whereas, tadalafil nanocrystals were dramatically smaller and more uniform than unprocessed particles. The nanoparticles were relatively spherical in shape with some degree of agglomeration that could be related to the drying process (Figure 4b).

*FTIR analysis*

FTIR analysis was used to evaluate the possible intermolecular interactions between tadalafil and the excipients and molecular structures of unprocessed drug, optimized nanocrystals and Tween 80 were examined through FTIR spectroscopy from 400 to 4000 cm^-1^. The main characteristic peaks of raw tadalafil were 751 cm^-1^ (benzene), 1037 cm^-1^ (C-O-C stretching), 1432 cm^-1^ (C-N stretching), 1644 cm^-1 ^(C=C aromatic), 1688 cm^-1 ^(C=O), 2901 cm^-1^ (C-H stretching) and 3322 cm^-1^ (N-H stretching). Moreover, four peaks at about 1110 cm^-1^ (C-O stretching), 1723 cm^-1^ (C=O stretching), 2932 cm^-1^ (C-H stretching) and 3390 cm^-1^ (O-H stretching) were found in FTIR spectra of Tween 80. FTIR spectra of the raw tadalafil and the tadalafil nanocrystals were similar in peak pattern and frequency and it could be deduced that there was no change in the chemical structure of drug and no interaction between drug and surfactant. 

*DSC analysis*

The changes in polymorphism and crystallinity of tadalafil before and after the process were assessed by comparing the DSC thermogram (Figure 6) and XRPD patterns (Figure 7) of all samples. The DSC data for raw tadalafil and nanoparticles are summarized in Table 4 and the DSC thermogram of raw tadalafil and tadalafil nanoparticles showed a peak at 301.03 °C and 292.96 °C, corresponded to the melting point of tadalafil. As shown in Figure 8, the melting point and enthalpy of tadalafil nanoparticles was decreased compared to those of raw tadalafil. The shifts towards lower values could be the result of reduction in size and/or indicates a decreased crystallinity for nanoparticles in the presence of Tween 80. In DSC thermogram of tadalafil nanoparticles, an endothermic peak in 29.96 °C is corresponding to the Tween 80. The presence of endothermic peak of tadalafil in both samples indicates that the drug is in the crystalline form which is good in the terms of physical stability.

*XRPD analysis*

X-ray diffraction was performed to determine the crystalline structure of particles. It was confirmed that no crystalline change was found in the nanoparticles, because their powder X-ray diffraction patterns were consistent with the pattern of the raw tadalafil (Figure 7). The characteristic peaks of drug exhibited in 2 *θ* of 7, 10.52, 12.45, 13.40, 14.43, 15.53, 16.86, 18.35, 21.59, 24.10 and 24.91. However, the difference in the relative intensities of peaks could be the result of size reduction and/or differences in the crystallinity of the samples. The results of XRPD analysis are in agreement with DSC results.

*Dissolution rate study*

Dissolution profiles and the data of dissolution efficiency (DE %) in 30 and 90 min for raw tadalafil and tadalafil nanocrystals are exhibited in Figure 8 and Table 5, respectively. Tadalafil nanocrystals reached up to 11.75 % and 16.50 % drug dissolution within 30 and 90 min, respectively. However, only 4.13% and 4.78% of raw tadalafil dissolved during the same period. The results indicated that tadalafil nanocrystals exhibited better dissolution property than the raw tadalafil. According to the Noyes–Whitney equation, the drug dissolution rate is directly proportional to the surface area exposed to the dissolution medium. The accelerated dissolution rate of tadalafil nanocrystals could be mainly attributed to their greater surface (35, 36). Recently, Krupa *et al.* (37) attempted to improve dissolution of tadalafil by its co-processing with the hydrophilic polymer Soluplus^®^ and the use of two methods: high energy ball milling and supercritical carbon dioxide impregnation (scco_2_). Finally, they reached to the highest dissolution amount of tadalafil, about 1.8fold in 120 min, as compared to unprocessed tadalafil that is fewer than obtained result by sonoprecipitation technique. In another study, Wlodarski *et al.* (38) revealed a significant, 20-fold, dissolution rate enhancement for the tadalafil vinylpyrrolidone-vinyl acetate co-polymer solid dispersion in comparison with crystalline tadalafil that it shows this technique could be as a more effective method for enhancing the dissolution rate of tadalafil.

*Saturation solubility*

Saturation solubility test for each tadalafil sample was performed over 48 h and the results of this test are shown in Figure 9. The final solubility of raw tadalafil in deionized water and phosphate buffer (pH 7.4) was 2.33 ± 0.06 µg/mL and 2.06 ± 0.03 µg/mL, whereas, that of the tadalafil nanocrystals were 8.06 ± 0.05 µg/mL and 5.73 ± 0.04 µg/mL, respectively. The saturation solubility of tadalafil nanocrystals in deionized water and phosphate buffer was 3.46 and 2.78 times more than raw tadalafil in these medium. The increase of solubility of tadalafil nanocrystals could be interpreted by particle size reduction.

## Conclusion

Tadalafil nanocrystals were successfully prepared using sonoprecipitation technique by applying acetone-DMSO as solvent, deionized water as antisolvent and Tween 80 as a stabilizer. The particle size of nanosuspensions was highly dependent on process parameters. The experimental design software successfully could determine the optimal conditions to achieve the desired response. Drastic enhancement of saturation solubility and dissolution rate was achieved by the reduction in the particle size of tadalafil. Sonoprecipitation technique can thus be a simple and effective approach to scale up and produce tadalafil nanocrystals in the industry to utilize in the form of tablet or inhalation drug delivery system.
